# Effectiveness of Outpatient Treatment in Ear, Nose, and Throat Clinics for Dysphagia and the Role of Questionnaires

**DOI:** 10.7759/cureus.66369

**Published:** 2024-08-07

**Authors:** Takafumi Yamano, Shoichi Kimura, Kaori Wada, Fumitaka Omori, Ayumi Nakamura, Kazumasa Fukuyo

**Affiliations:** 1 Section of Otorhinolaryngology, Department of Medicine, Fukuoka Dental College, Fukuoka, JPN; 2 Department of Otorhinolaryngology, Fukuoka Dental College Hospital, Fukuoka, JPN; 3 Department of Physical Medicine and Rehabilitation, Fukuyo ENT Clinic, Itoshima, JPN; 4 Department of Otolaryngology, Fukuyo ENT Clinic, Itoshima, JPN

**Keywords:** and throat clinics, nose, ear, swallowing, dysphagia, questionnaire, fiberoptic endoscopic evaluation of swallowing

## Abstract

Background

While most research on dysphagia treatment has focused on inpatients, less attention has been given to outpatient settings, particularly in ear, nose, and throat (ENT) clinics. Additionally, while questionnaires are commonly used as screening tools in dysphagia management, their correlation with outcomes such as pneumonia incidence or sustained oral intake is rarely discussed. This study aimed to evaluate the effectiveness of outpatient treatment in ENT clinics for dysphagia, including improvement in subjective symptoms, and to assess the role of the questionnaire.

Methodology

In total, 59 patients (38 males and 21 females) aged 53-93 years (mean age = 79 years) attended the outpatient swallowing clinic. All participants retained sufficient ability in activities of daily living to independently visit the hospital and could orally ingest food, and none required tube feeding. Subjective symptoms were evaluated using the questionnaire. Swallowing assessments were conducted by an otolaryngologist and via swallowing endoscopy. A speech-language pathologist led the swallowing rehabilitation, which included encouraging family involvement and home practice.

Results

The most frequent issue reported was munching during meals. Of the 59 patients, 22 underwent continuous outpatient rehabilitation. Of these, 17 (77%) showed improvement; 11 had improvement in both subjective symptoms and fiberoptic endoscopic evaluation of swallowing (FEES) scores, five in subjective symptoms only, and one in FEES scores only. Five patients showed no change/worsening conditions.

Conclusions

The questionnaire proved useful as a screening tool but fell short in terms of prognosis estimation. The findings suggest that information from the questionnaire should be used to gauge treatment effectiveness, noting that some cases showed improvement in subjective symptoms alone.

## Introduction

Most studies on dysphagia treatment have centered on inpatients in acute and convalescent hospitals, primarily addressing cerebrovascular disorders and degenerative diseases. There has been little focus on outpatients, particularly in otorhinolaryngology clinics. At our institution, we operate a specialized weekly outpatient clinic for swallowing disorders, providing treatment primarily for minor cases, with notable success [1]. Moreover, although questionnaires are widely reported as useful screening tools in dysphagia treatment, their correlation with prognostic factors such as pneumonia frequency or sustained oral intake is seldom discussed. This study aims to evaluate the effectiveness of outpatient dysphagia treatment in an ear, nose, and throat (ENT) clinic, including improvements in subjective symptoms and the use of the questionnaire.

## Materials and methods

Outpatient swallowing system

**Figure 1:**
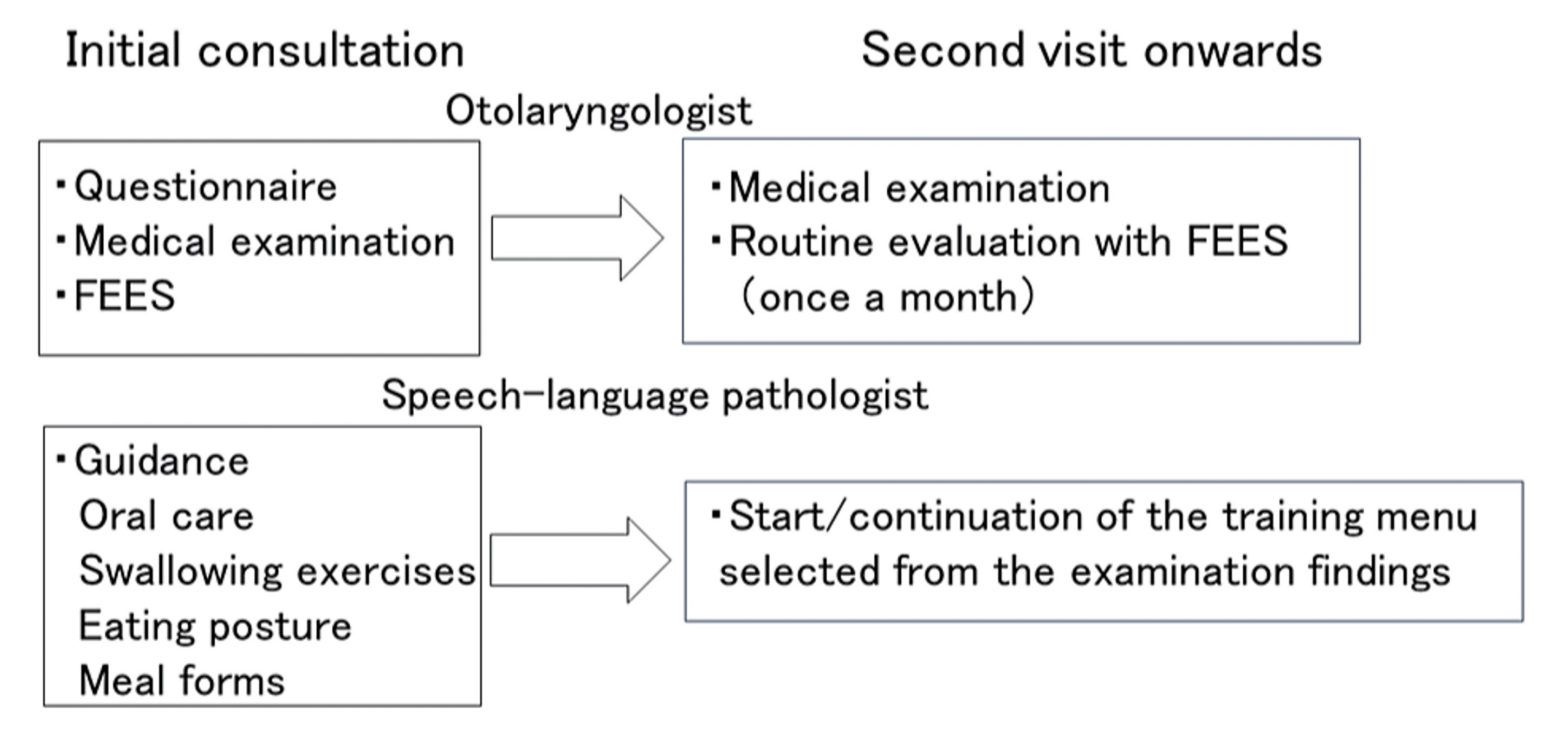
Flow of outpatient consultations. FEES: fiberoptic endoscopic evaluation of swallowing

The outpatient consultation process is shown in Figure 1.

The team consisted of an otolaryngologist and a speech-language pathologist. During the initial visit, patients completed a questionnaire, and the otolaryngologist conducted an observation of the pharynx and swallowing endoscopy. Subsequent visits involved monthly assessments by the ENT doctor using swallowing endoscopy, while the speech-language pathologist began and then continued rehabilitation based on the examination results.

Patients

Between June 2022 and December 2023, the Fukuyo ENT Clinic attended to 59 patients (38 males and 21 females) aged 53-93 years (mean age = 79 years). All participants retained sufficient ability in activities of daily living (ADLs) to independently visit the hospital and could orally intake food, and none required tube feeding. All cases seen during the study period were included and no cases were excluded.

Questionnaire

The questionnaire, adapted from Ohkuma et al. [2], comprised the following 15 questions: Q1: Have you ever been diagnosed with pneumonia? Q2: Do you feel you are becoming thin? Q3: Do you ever have difficulty when you swallow? Q4: Do you ever choke during meals? Q5: Do you ever choke when swallowing liquids? Q6: Do you ever have difficulty coughing up phlegm during or after a meal? Q7: Do you ever have the feeling that food is getting stuck in your throat? Q8: Does it take you longer to eat a meal than before? Q9: Do you feel that it is getting difficult to eat solid foods? Q10: Do you ever drop food from your mouth? Q11: Do you ever have the feeling that food is remaining in your mouth? Q12: Do you ever have the feeling of food or liquid going up into your throat from your stomach? Q13: Do you ever have the feeling that food is getting stuck in your esophagus? Q14: Do you ever have difficulty sleeping because of coughing during the night? Q15: Do you feel that your voice is getting hoarse? Each item was rated on a three-point ABC scale, where the severity of the complaints was in the order A > B > C (Table 1).

**Table 1:** The Ohkuma questionnaire.

Number	Question	Answer		
1	Have you ever been diagnosed with pneumonia?	A: Many times	B: Once	C: No
2	Do you feel you are becoming thin?	A: Obviously	B: Slightly	C: No
3	Do you ever have difficulty when you swallow?	A: Many times	B: Sometimes	C: No
4	Do you ever choke during a meal?	A: Many times	B: Sometimes	C: No
5	Do you ever choke when swallowing liquids?	A: Many times	B: Sometimes	C: No
6	Do you ever have difficulty with coughing up phlegm during or after a meal?	A: Many times	B: Sometimes	C: No
7	Do you ever have the feeling that food is getting stuck in your throat?	A: Many times	B: Sometimes	C: No
8	Does it take you longer to eat a meal than before?	A: Obviously	B: Slightly	C: No
9	Do you feel that it is getting difficult to eat solid foods?	A: Obviously	B: Slightly	C: No
10	Do you ever drop food from your mouth?	A: Many times	B: Sometimes	C: No
11	Do you ever have the feeling that food is remaining in your mouth?	A: Many times	B: Sometimes	C: No
12	Do you ever have the feeling of food or liquid going up into your throat from your stomach?	A: Many times	B: Sometimes	C: No
13	Do you ever have the feeling that food is getting stuck in your esophagus?	A: Many times	B: Sometimes	C: No
14	Do you ever have difficulty sleeping because of coughing during the night?	A: Many times	B: Sometimes	C: No
15	Do you feel that your voice is getting hoarse?	A: Obviously	B: Slightly	C: No

Fiberoptic endoscopic evaluation of swallowing

An otolaryngologist conducted the fiberoptic endoscopic evaluation of swallowing (FEES) using Hyodo’s score [3]. Four aspects were assessed on a four-point scale, namely, salivary retention in the epiglottis valley and pear-shaped depression, responsiveness of the cough reflex and glottal closure reflex, responsiveness of the swallowing reflex, and pharyngeal clearance upon swallowing 3 cc of colored water (0-3, respectively).

Rehabilitation

A speech-language pathologist led the rehabilitation, focusing on oral care, instruction in food morphology and eating methods, compensatory swallowing techniques, expectoration training (abdominal breathing, huffing, vocal exercises), oral swallowing organ function exercises, articulation training, environmental adjustments, and physical fitness recommendations. Each session lasted ~30 minutes, with family members encouraged to attend. Patients were also encouraged to practice independently at home where possible.

Statistical analysis

Data analysis was conducted using the Mann-Whitney U test.

## Results

Breakdown of subjective symptoms

**Figure 2:**
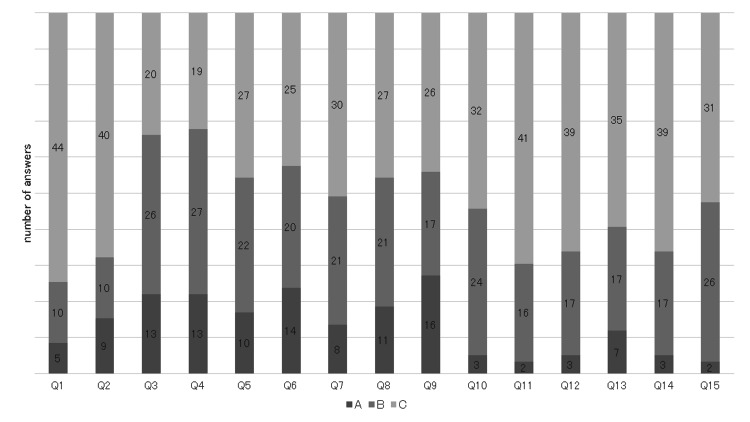
Breakdown of perceived symptoms.

Details of subjective symptoms are shown in Figure 2.

Q1 had 5 cases of A, 10 cases of B, and 44 cases of C; Q2 had 9 cases of A, 10 cases of B, and 40 cases of C; Q3 had 13 cases of A, 26 cases of B, and 20 cases of C; Q4 had 13 cases of A, 27 cases of B, and 19 cases of C; and Q5 had 10 cases of A, 22 cases of B, and 27 cases of C. The most common symptoms, receiving A and B ratings, were Q4, indicating choking during meals, followed by Q3, reflecting general difficulty in swallowing, and Q5, related to issues swallowing liquids.

Effects of the intervention

**Figure 3:**
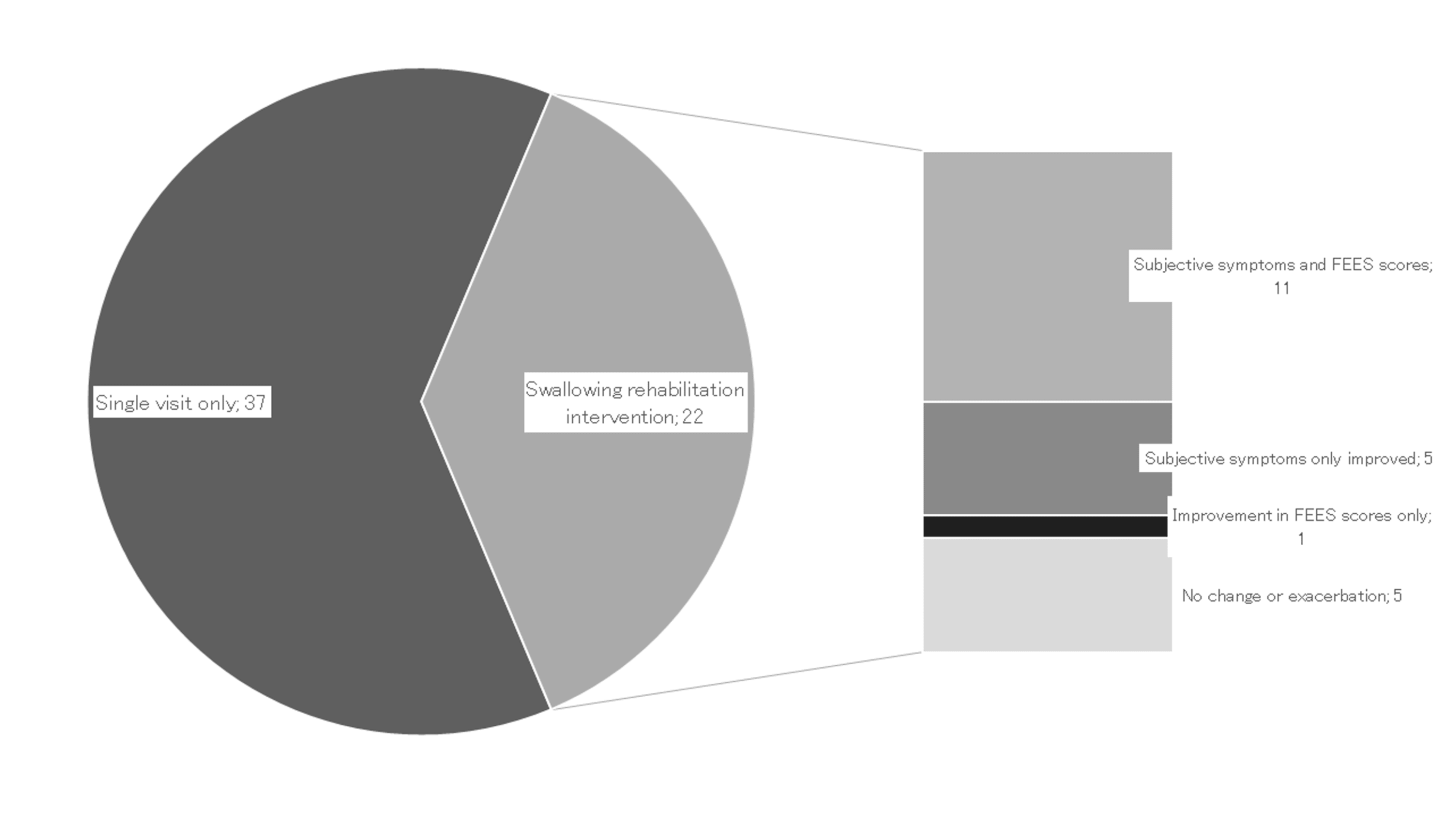
Effectiveness of the intervention. FEES: fiberoptic endoscopic evaluation of swallowing

Of the 59 patients, 37 attended only one visit while 22 participated in ongoing outpatient rehabilitation. Among those 22 patients, outcomes were categorized as improved, unchanged, or worsened. Improvement was defined as any positive change in at least one item. Both subjective symptoms and fiberoptic endoscopic findings improved in 11 cases, subjective symptoms alone improved in five cases, fiberoptic endoscopic findings alone improved in one case, and five cases were unchanged or worsened (Figure 3).

Comparison of questionnaire items between improved and no change/worsened cases

Of the 22 patients who received outpatient rehabilitation, 17 were classified as improving and five as unchanged or worsening, except for one patient whose FEES only improved. When the answers to the initial medical interview were compared, no statistically significant differences were found in 14 items. Only in question 11, indicating acid reflux symptoms, the unchanged or worsened cases complained more strongly than the improved cases (p = 0.019) (Table 2).

**Table 2:** Comparison of questionnaire items between improved and no change/worsened cases. Only in question 11, indicating acid reflux symptoms, the unchanged or worsened cases complained more strongly than the improved cases (p = 0.019).

	Improvement (N = 16)			No change/Worsened cases (N = 5)		
	A	B	C	A	B	C
Q1	2	3	11	0	2	3
Q2	3	0	13	1	2	2
Q3	3	5	8	2	2	1
Q4	0	11	5	1	4	0
Q5	0	9	7	1	3	1
Q6	4	7	5	2	2	1
Q7	1	6	9	2	2	1
Q8	2	6	8	2	1	2
Q9	4	5	7	0	4	1
Q10	0	4	12	0	4	1
Q11	0	3	13	1	2	2
Q12	1	4	11	1	4	0
Q13	1	6	9	1	0	4
Q14	0	6	10	0	1	4
Q15	0	5	11	0	3	2

Details of no change/worsened cases

The details of the five cases that showed no change/worsened status are outlined in Table 3.

**Table 3:** Details of no change/worsened cases. FEES: fiberoptic endoscopic evaluation of swallowing

Age	Gender	Primary disease	Length of hospital stay	A	B	C	FEES scores
81	Male	Laryngeal cancer radiotherapy case	176	1	8	6	5
71	Male	Sequelae of cerebral infarction/Dementia	56	6	6	3	4
72	Male	Amyloidosis	209	1	8	6	4
84	Male	Sleep apnea syndrome/Sequelae of cerebral infarction	84	0	5	10	3
83	Male	Dementia/Reflux esophagitis/Interstitial pneumonia	49	7	8	0	8

These patients were all males aged 71-84 years. Their complications included irreversible dysphagia conditions such as post-laryngeal cancer radiotherapy, amyloidosis, and interstitial pneumonia, as well as disorders involving acid reflux such as sleep apnoea and reflux esophagitis. The length of the outpatient treatment period ranged from 49 to 209 days, and there was variability in the degree of subjective symptoms and swallowing endoscopy scores among these individuals.

## Discussion

Most studies on questionnaires used in dysphagia practice highlight their role as screening tools. The widely used Eating Assessment Tool-10 (EAT-10) features 10 questions rated from 0 (no problem) to 4 (severe problem), with scores of ≥3 indicating suspected impaired swallowing function [4]. A study involving the same demographic as the present one (non-institutionalized older adults) found that 30% scored ≥3, demonstrating its efficacy as a screening test [5].

Uhm et al.’s Easy Dysphagia Symptom Questionnaire integrates a revised water drinking test and the American Speech-Language-Hearing Association’s National Outcome Measurement System swallowing scale, and is correlated with the Videofluoroscopic Dysphagia Scale [6]. The Dysphagia in Multiple Sclerosis questionnaire, initially designed for dysphagia assessment in multiple sclerosis patients [7], has recently been shown to be effective in assessing Parkinson’s disease as well [8]. The Ohkuma questionnaire, used in this study, was first used to identify dysphagia in Japanese nursing home residents [2] and proved to be an effective screening tool for community-dwelling older adults aged ≥65 years, showing significant differences in ability scores, stroke history, and health perceptions between those with and without dysphagia [9]. Additionally, its translations into Chinese [10] and Greek [11] have also been recognized as valuable tools for identifying poor swallowing function.

However, reports linking questionnaire results to prognosis are scarce: neither the severity of dysphagia, as assessed by the questionnaire of Ohkuma et al., nor the EAT-10 correlated with the subsequent onset of pneumonia [12]. In this study, significant differences were found only in acid reflux items when comparing improved cases with unchanged or worsening cases, with no other significant prognostic correlations. Therefore, while the questionnaire proved useful for initial screening, its capacity to predict prognosis remains limited.

Regarding the effect of outpatient rehabilitation for dysphagia, we previously reported improvements in tongue pressure and a significant reduction in salivary retention in the glottis valley and pear-shaped depression, as assessed by FEES [1]. Other centers have detailed more structured, protocol-based interventions, with one report showing a 78.8% improvement in pharyngeal phase swallowing among patients with resistant hypertensive disease and obstructive sleep apnea syndrome [13], along with a similar improvement in swallowing metrics such as the Mann Assessment of Swallowing Ability, Functional Oral Intake Scale, visual analog scale, hyolaryngeal excursion, lingual-palatal pressure, and surface electromyography assessments, noting a sustained effect after five months of training [14]. Conversely, a study on patients with Parkinson’s disease highlighted variable effectiveness, assessed using swallowing contrast [15], with results differing based on the targeted condition. In the current study, 17 out of 22 patients (77%) who continued outpatient rehabilitation for mild dysphagia reported improvement. This figure includes cases with only subjective symptom improvements, suggesting the questionnaire data should be interpreted as one aspect of efficacy.

This study had some limitations. It only included patients well enough to attend outpatient sessions and excluded those with severe conditions prohibiting oral intake. Additionally, variability in underlying diseases precluded detailed analysis of the severity and characteristics of each condition. Furthermore, the 15 questionnaire items covered a range of swallowing stages, from mastication to esophageal swallowing, indicating a need for future studies to align better with specific swallowing pathologies and stages.

## Conclusions

In the outpatient treatment of minor cases at an ENT clinic, the questionnaire was useful as a screening tool, but it was challenging to infer prognosis. The improvement in 17 of 22 cases (77%) was primarily in subjective symptoms, suggesting that the information from the questionnaire should be considered when assessing efficacy.
